# Emergency contraception among Finnish adolescents: awareness, use and the effect of non-prescription status

**DOI:** 10.1186/1471-2458-7-201

**Published:** 2007-08-09

**Authors:** Kobra Falah-Hassani, Elise Kosunen, Rahman Shiri, Arja Rimpelä

**Affiliations:** 1 Tampere School of Public Health, University of Tampere, Tampere, Finland; 2 Tampere University Hospital, Tampere, Finland; 3 Medical School, University of Tampere, Tampere, Finland; 4 National Research and Development Centre for Welfare and Health (STAKES), Finland

## Abstract

**Background:**

Adolescents need to be aware that there is a method of preventing pregnancy even after an unprotected intercourse. Limited information is available on the awareness of young adolescents and the effects of selling emergency contraception (EC) over-the-counter, and the findings are controversial. The aims of this study were to investigate awareness and use of EC among Finnish girls aged 12–18 years in 1999–2003, and to assess the effect of the 2002 non-prescription status on the use.

**Methods:**

A self-administered questionnaire was sent to a population-based sample of 12–18-year-olds girls in 1999, 2001, and 2003. Response rate was 83% in 1999 (N = 4,369), 79% in 2001 (N = 4,024) and 77% in 2003 (N = 3,728), altogether N = 12,121. Logistic regression model was used to examine the association of unawareness and use of EC with socio-economic background and health behaviour.

**Results:**

In 2001, nearly all 14–18-year-olds and a majority of 12-year-olds were aware of EC. Among 12–14-year-olds, a slight increase in awareness between 1999 and 2003 was observed but this was not related to non-prescription status. Health-compromising behavior (alcohol use, smoking), dating and having good school achievement were related to higher awareness of EC.

Nine percent of 14–18-year-olds had used EC once and 1% three times or more. No statistically significant change in EC use was found after non-prescription status. EC use increased with increasing alcohol consumption, particularly at age 14. Smoking, dating, and poor school achievement were related to increased use as well as not living in nuclear family. A lower use was observed if living in rural area or father's education was high. Mother's education was not related to use.

**Conclusion:**

Adolescent girls were well aware of the existence of emergency contraception even before the non-prescription status. Over-the-counter selling did not increase the use.

## Background

The availability of emergency contraception is important for adolescents in particular. A condom is the most frequently used contraceptive method in early stages of sexual activity, but also failures in the use are frequent. In addition, completely unprotected sexual encounters are more usual in early years of sexual career than in adult age [1].

Hormonal emergency contraception (EC) has been known for over three decades. The first commercial product contained both estradiol and levonorgestrel (Yuzpe method). Because of the potential side effects of the estrogen component, a doctor's prescription was required for obtaining it. After the levonorgestrel-only method was introduced on the market, and showed to be more effective with less side-effects compared to the Yuzpe method [2], many countries accepted selling it over-the-counter without a prescription in order to improve availability. In Finland, over-the counter sales to those aged 15 or older was accepted in 2002. Adolescents do not need parental consent for buying EC from pharmacies and access to family planning services is confidential.

Limited information is available on the effects of selling EC over-the-counter, and the findings are controversial. A study among girls aged 16 to 19 years in UK concluded that over-the-counter sales had no effect on the use of EC [3] while a study in Canada among girls aged 15–19 years showed an increase [4]. In Finland, sale figures of EC products increased 62% in 2002 when EC change to non-prescription status was carried out [5]. No detailed analysis of the effects of the change in prescription status or on socioeconomic and regional distribution in the adolescent EC use has been published.

Among sexually active girls aged 14–20 years the life-time use of EC has ranged from 10% in England to 28% in Sweden [6-11]. In Finland, 6.6% of all girls aged 14–17 years had used EC in 1996 [9]. Most studies on the EC use have been carried out under circumstances where the product was not available without a doctor's prescription. Under such circumstances, the use of EC reflects to a great extent how easy the access to services is. It is well known that access to services has a strong impact on adolescent contraceptive use.

To be able to seek EC, adolescents need to be aware that there is a method of preventing pregnancy even after an unprotected intercourse. The awareness of EC in adolescent population has been studied in some countries. The prevalence has varied a great deal, from 28% in the USA to 81% in the UK [6,8,10-14]. A few studies have also shown that even if adolescents were aware of EC, only a small proportion knew of the time limits within which EC is effective [8,10,12,14]. In Finland, the first study on awareness of EC in 1996 showed that over 90% of 14–16-year-old adolescents knew about the method [9].

Early sexual activity is related to many factors reflecting risk-taking life-style like having multiple sex partners, smoking and drinking [6,15]. There is limited information available on the associations between life-style factors and EC use in adolescence. In the Swiss study, EC use was higher in girls with more than three partners, first love affair before 14 years, regular sexual intercourse, unplanned or unconscious first intercourse and a history of pregnancy [6]. Teenagers who consumed alcohol took EC more frequently than non-consumers [16]. Alcohol intake is often connected with unplanned, unprotected intercourse [15,17].

In this study, we investigate the use and awareness of the emergency contraception as well as their variation by individual and family factors and place of residence, among Finnish girls aged 12–18 years. The impact of allowing easier access to the product through over-the-counter status is studied by comparing the years 1999–2001 with the year 2003.

## Methods

### Participants

In the Adolescent Health and Lifestyle Survey (AHLS), data have been collected every second year since 1977 by postal inquiry to national samples of 12, 14, 16 or 18 years residing in Finland. The use of emergency contraceptive was asked in 1999, 2001 and 2003. The cover letter mentions that if parents want to see the questionnaire, they should do so before answering. The samples were obtained from the population register centre and based on selected dates of birth (July in some years and dates from June and August included too), so that all Finns born on the given days were included. Self-administered questionnaires were mailed in February with two re-inquiries to non-respondents. The timing of the study, sampling and data collection methods were similar throughout the study period.

Response rate was 83% in 1999 (N = 4,369), 79% in 2001 (N = 4,024) and 77% in 2003 (N = 3,728). Subjects (N = 208) with missing information on emergency contraception were excluded and 11,913 girls aged 12, 14, 16 or 18 years included in the study. For the analyses of the characteristics of emergency contraceptive use, girls aged 12 years were excluded because none of them used EC and finally 10,899 were included.

The AHLS study protocol has been approved by the ethical committee of the Department of Public Health of the University of Helsinki.

### Measures

The awareness and use of emergency contraceptive was investigated with the question: Have you ever used emergency contraceptive? The responses alternatives were:

• I do not know what emergency contraception is

• No, I have not used

• Yes, how many times altogether______

The outcomes of the study were the unawareness (or awareness) and use of emergency contraception. Unawareness (awareness) was defined as not knowing (knowing) what emergency contraception is and EC use defined as taking it at least once.

### Background characteristics

Background characteristics were divided into three groups; individual, family factors and factors describing the place of residence.

#### Individual factors

*Educational career *for girls aged 16 or 18 years was classified in: not in school, vocational school or high school. *School achievement *based on pupil's own assessment of his or her position in class according to latest report was categorized: much better and slightly better (than average), average, and poorer (than average). The majority of 16 and 18-year-old girls were still in school, and nearly 90% reported their latest school achievement in this age group. *Daily smoking *was defined as using tobacco every day. Information on the frequency of *alcohol consumption *was asked by a question: How often do you use alcohol? The answers were classified: 1) never 2) less than monthly 3) monthly and 4) weekly. *Dating *was a dichotomized variable.

#### Family factors

*Fathers' education *as well as *mother's education *was classified into three categories: low, middle and high. Variable *family structure *describes people living in the participant's family (mother and father, mother or father and a stepparent, one-parent family, husband/partner, other guardian). Open question on *father's occupation *was categorized into upper white collar, lower white collar, farmer or forestry, and blue collar.

#### Factors describing the place of residence

The country was divided into four geographical *regions*: south, north, east and west. The *urbanization level *of the place of residence was defined by population density: capital city area (the capital Helsinki and the adjoining towns), other towns, villages in rural municipality, and sparsely populated rural areas (isolated homesteads in rural municipalities).

### Statistical methods

Statistical significance (two-tailed *p*-value < 0.05) for a linear trend in the proportion of emergency contraceptive awareness or use was used in untransformed data. Logistic regression models were run to study the characteristics of emergency contraceptive awareness and use in the data set emerging all three survey years. To perform efficiently a logistic regression model, unawareness of emergency contraception was used as an outcome of interest because the proportion of girls aware of EC was very high.

Odds ratios (OR) and 95% of confidence intervals (CI) of the unawareness and use according to background characteristics were estimated. First, age and survey year adjusted models were fitted separately for each explanatory variable after excluding cases with missing data for the variable concerned. Second, all background variables significant (<0.20) in the previous models were included in the multivariate models.

Finally, logistic regression models were run separately for prescription (1999–2001) and non-prescription (2003) periods in order to see whether over-the-counter status had any effect on variation of the awareness or use. For the unawareness, the models were run separately for 12–14 and 16–18 years old.

To study repeatability of the questions, a sub-sample of 14- and 16-year-olds (n = 407) was randomly selected from the original subject series of 2003. Of these, 327 (80%) girls had responded to the inquiry. An identical questionnaire was mailed to these 327 girls approximately four weeks after receipt of the original one; 274 of them (67%) returned the second questionnaire. Test-retest reliability of EC use was tested with κ-coefficient. The κ was .74, which indicates a substantial agreement beyond chance between the two questionnaires.

For the analysis estimation of the effects of non-respondents, the data was divided into three categories according to the return date of the questionnaire. It was assumed that the later a person answers (original questionnaire/first re-inquiry/second re-inquiry) more she resembles as a non-respondent. EC use was 10.8% among 8,761 respondents to original questionnaire, 13.6% among 2,429 respondents to first re-inquiry and 16.0% among 723 girls who returned the second re-inquiry. There was no statistically significant difference in EC use among girls aged 14 years (p = 0.37) whereas EC use was higher in late-respondents aged 16 (p = 0.007) or 18 years (p = 0.003).

## Results

### Prevalence and trend

#### Awareness of emergency contraception

In 2003, 61% of girls aged 12 years knew about EC and 98% of those aged 18 (Table 1). Among 16–18-year-olds, the awareness did not change between 1999 and 2003. Among 12-year-olds the increase in the awareness was seen before the non-prescription status in 2001, and in 14-year-olds, a small increase was seen both before and after.

**Table 1:** Age-specific percentage of adolescents who were aware of emergency contraception and those who had used EC according to the year of survey

Emergency contraception	Prescription status for EC		Non-prescription status for EC	N	*p*-value *
				
	1999	2001	2003		
Awareness of EC					
Age					
12	58	67	61	1164	0.02
14	91	94	96	3966	<0.001
16	98	99	99	3732	0.18
18	99	99	98	3051	0.29
					
EC use					
Age					
14	2	3	2	3966	0.22
16	13	13	15	3732	0.24
18	24	26	29	3051	0.09

* Test for trend

#### Emergency contraception use

In 2003, 2% of girls aged 14 years had ever used EC. For those aged 16 and 18, the percentages were 15% and 29% (Table 1). No statistically significant increase in EC use was found in any of the age groups over time. One year after the change to non-prescription status, there was a slight increase from 13% to 15% in girls aged 16. The corresponding figures were 24% and 29% for those aged 18, but the increasing trend existed already before the change in non-prescription status.

### Characteristics

#### Unawareness of emergency contraception

Among girls aged 12–14 years, EC unawareness was associated with alcohol consumption, smoking, dating, school achievements and urbanization level, but not with socio-economic background and region (Table 2). EC unawareness was lower among alcohol drinkers, smokers and those who had a dating relationship, and were higher in girls with school achievements much better than average. Girls from rural villages or sparsely populated areas were less often unaware of EC than those from capital city area.

**Table 2:** The proportion and odds ratio (OR) of unawareness of emergency contraceptive according to background characteristics in girls aged 12–14 years

Background characteristic	Sample	% of unawareness	Adjusted for age and survey year			Multivariate *	
				
			OR	95%	P value	OR	95% CI
*Individual factors*							
Alcohol consumption							
Not drinking	2243	22.0	1			1	
Less than monthly	1386	7.9	**0.6**	**0.4–0.7**		**0.4**	**0.3–0.6**
Monthly	979	3.4	**0.3**	**0.2–0.4**		**0.3**	**0.2–0.4**
Weekly	360	3.9	**0.3**	**0.2–0.6**	<0.001	**0.3**	**0.1–0.6**
							
Daily smoking							
No	4462	14.5	1			1	
Yes	557	3.8	**0.5**	**0.3–0.8**	0.005	0.9	0.5–1.6
							
Dating							
No	4428	14.5	1			1	
Yes	620	5.0	**0.5**	**0.3–0.8**	0.001	0.9	0.5–1.4
							
School achievements							
Much better than average	864	10.4	1			1	
Slightly better than average	1667	13.4	1.2	0.9–1.6		**1.7**	**1.1–2.5**
Average	1979	15.0	**1.4**	**1.1–1.8**		**2.2**	**1.5–3.3**
Poorer	507	10.8	1.4	0.9–2.1	0.01	**2.2**	**1.3–3.9**
							
*Family factors*							
Family structure							
Mother-father	3872	13.2	1				
One parent and one stepparent	480	15.4	1.2	0.9–1.6		..	..
One-parent family	690	12.0	1.0	0.7–1.2			
Other	21	14.3	1.0	0.3–3.8	0.94		
							
Father's education							
Low	898	13.8	1				
Middle	2598	13.5	0.9	0.7–1.2		..	..
High	1196	12.5	0.9	0.7–1.2	0.52		
							
Father's occupation							
Upper white collar	1773	12.4	1				
Lower white collar	1147	14.1	1.3	0.9–1.6		..	..
Farmer or forestry	317	13.6	1.1	0.7–1.6			
Blue collar	1747	13.1	1.1	0.9–1.3	0.82		
							
Mother's education							
Low	606	14.4	1				
Middle	2381	13.2	0.8	0.6–1.0		..	..
High	1812	13.5	0.8	0.6–1.1	0.52		
							
*Factors describing the place of residence*							
Urbanization level							
Capital city area	704	14.9	1			1	
Cities	2435	13.8	0.8	0.6–1.1		0.9	0.6–1.3
Rural villages	1098	11.3	**0.7**	**0.5–0.9**		0.7	0.5–1.1
Sparsely populated areas	751	13.4	**0.7**	**0.5–0.9**	0.03	0.7	0.4–1.1
							
Regions							
Southern	1853	14.4	1			..	..
Northern	802	13.0	0.8	0.6–1.0			
Eastern	661	12.1	0.8	0.6–1.0			
Western	1814	12.8	0.8	0.7–1.0	0.10		

Statistically significant ORs are bolded

*Adjustment for age, survey year, age at menarche and the significant variables of the previous model.

.. Not included in the multivariate model

In multivariate analysis controlled for all covariates significant in previous model as well as age and survey year, only alcohol consumption and school achievement were statistical significantly associated with the unawareness (Table 2). EC unawareness decreased with increment in the level of alcohol consumption. Compared to girls whose school performance was much better than average, the unawareness of EC was higher in girls whose school achievements were slightly better than average, average, or poorer than average.

Among girls aged 16–18 years, EC unawareness was less among alcohol consumers, smokers, those with dating relationship or whose mothers had high education, girls at high school or lived in cities or rural villages (Table 3). The lack of knowledge was high in girls living with guardians other than their own parents, and was lower in the eastern than in the southern of Finland. Family structure, father's occupation, father's education and the level of urbanization were not related to EC unawareness.

**Table 3:** The proportion and odds ratio (OR) of unawareness of emergency contraceptive for background characteristics in girls aged 16–18 years

Background characteristic	Sample	% of unawareness	Adjusted for age and survey year			Multivariate *	
				
			OR	95%	P value	OR	95% CI
*Individual factors*							
Alcohol consumption							
Not drinking	658	5.2	1			1	
Less than monthly	1716	1.3	**0.2**	**0.1–0.4**		**0.3**	**0.1–0.6**
Monthly	2677	0.4	**0.1**	**0.04–0.2**		**0.1**	**0.05–0.3**
Weekly	1582	0.7	**0.1**	**0.06–0.3**	<0.001	**0.1**	**0.03–0.3**
							
Daily smoking							
No	4614	1.4	1			1	
Yes	2055	0.6	**0.4**	**0.2–0.8**	0.007	0.5	0.2–1.1
							
Dating							
No	3921	1.5	1			1	
Yes	2790	0.8	**0.5**	**0.3–0.9**	0.01	0.6	0.3–1.1
							
School achievements							
Much better than average	777	1.5	1				
Slightly better than average	1924	1.0	0.7	0.3–1.4		..	..
Average	2925	0.9	0.6	0.3–1.1			
Poorer	699	2.0	1.3	0.6–2.8	0.82		
							
Educational career							
Not in school	393	3.1	1			1	
Vocational school	1852	2.4	0.8	0.4–1.5		0.8	0.3–1.7
High school	4345	0.5	**0.2**	**0.1–0.3**	<0.001	**0.2**	**0.1–0.4**
							
*Family factors*							
Family structure							
Mother-father	4819	1.2	1			1	
One parent and one stepparent	572	1.0	0.9	0.4–2.0		0.7	0.2–2.5
One-parent family	989	1.5	1.3	0.7–2.3		1.5	0.8–3.0
Husband/wife or cohabiting	238	0.4	0.4	0.1–2.7		0.5	0.1–3.8
Other guardian	40	7.5	**6.8**	**2.0–22.8**	0.26	**6.9**	**1.8–26.7**
							
Father's education							
Low	1449	1.1	1				
Middle	3409	1.0	0.9	0.5–1.7		..	..
High	1523	1.2	1.1	0.5–2.1	0.80		
							
Father's occupation							
Upper white collar	2358	0.8	1				
Lower white collar	1493	1.3	1.5	0.8–2.8		..	..
Farmer or forestry	428	1.9	2.2	0.9–5.1			
Blue collar	2337	1.1	1.3	0.7–2.3	0.45		
							
Mother's education							
Low	1008	1.8	1			1	
Middle	3349	1.1	0.6	0.3–1.1		0.9	0.5–1.8
High	2139	0.8	**0.4**	**0.2–0.9**	0.02	0.8	0.3–1.7
							
*Factors describing the place of residence*							
Urbanization level							
Capital city area	890	2.4	1			1	
Cities	3669	1.1	**0.5**	**0.3–0.8**		0.5	0.2–1.2
Rural villages	1432	0.4	**0.2**	**0.1–0.4**		**0.2**	**0.1–0.7**
Sparsely populated areas	704	2.0	0.8	0.4–1.6	0.14	1.0	0.4–2.6
							
Regions							
Southern	2507	1.6	1			1	
Northern	1060	1.1	0.7	0.4–1.4		1.2	0.5–2.7
Eastern	825	0.5	**0.3**	**0.1–0.9**		0.4	0.1–1.3
Western	2391	1.2	0.8	0.5–1.3	0.19	1.1	0.5–2.3

Statistically significant ORs are bolded

*Adjustment for age, survey year, age at menarche and the significant variables of the previous model.

.. Not included in the multivariate model

In multivariate analysis controlled for all covariates significant in previous model as well as age and survey year, alcohol consumption, educational career and family structure remained statistically significant (Table 3). EC unawareness decreased with increment in the level of alcohol intake, and was less in girls who were at high school compared with those who were not at school at all. Teens living with guardians other than their own parents were more often unaware of EC than the teens lived with their parents. Girls living in rural villages had less often lack of knowledge than girls lived in the capital city.

Characteristics of EC unawareness were similar in prescription (1999–2001) and non-prescription (2003) periods.

#### Emergency contraception use

After adjustment for age and year of survey, the use of EC among girls aged 14–18 years increased with alcohol consumption, daily smoking and dating relationship (Table 4). EC use was higher in girls whose school achievements were average or poorer than average compared with those whose school achievements were much better than average. Girls who did not live with nuclear family used EC more frequently than girls living with both parents. However, girls having other guardians than own parent did not differ from girls with a nuclear family. EC intake was lower in teens whose fathers had high education relative to those whose fathers had low education. Girls whose fathers were farmers or forestry workers used EC less than other groups. Girls lived in cities used more often EC than those lived in the capital city. On the other hand, teens lived in sparsely populated areas used EC least often.

**Table 4:** The prevalence and odds ratio (OR) of emergency contraceptive use according to background characteristics in girls aged 14, 16 or 18 years

Background characteristic	Sample	%	Adjusted for age and survey year		Multivariate *	
				
			OR	95%	OR	95% CI
*Individual factors*						
Alcohol consumption						
Not drinking	1965	0.9	1		1	
Less than monthly	2952	7.8	**6.4**	**3.9–10.4**	**4.7**	**2.7–8.4**
Monthly	3622	16.2	**11.2**	**6.7–18.0**	**6.3**	**3.6–11.1**
Weekly	1936	27.6	**19.4**	**12.0–31.4**	**9.7**	**5.4–17.2**
						
Daily smoking						
No	7943	8.3	1			
Yes	2609	27.3	**3.4**	**3.0–3.8**	**2.0**	**1.7–2.4**
						
Dating						
No	7249	6.2	1		1	
Yes	3367	27.4	**3.8**	**3.3–4.3**	**3.1**	**2.6–3.5**
						
School achievements						
Much better than average	1464	8.3	1		1	
Slightly better than average	3180	10.8	1.2	0.9–1.5	1.1	0.8–1.4
Average	4418	14.0	**1.5**	**1.2–1.8**	1.1	0.9–1.5
Poorer	1148	17.0	**2.2**	**1.7–2.9**	1.3	0.9–1.8
						
*Family factors*						
Family structure						
Mother-father	7800	11.1	1		1	
One parent and one stepparent	939	16.3	**1.7**	**1.4–2.0**	**1.3**	**1.1–1.6**
One-parent family	1540	15.3	**1.4**	**1.2–1.7**	**1.2**	**1.0–1.5**
Husband/wife or cohabiting	246	38.2	**2.4**	**1.8–3.1**	1.2	0.8–1.7
Other guardian	48	20.8	1.4	0.7–2.9	1.5	0.6–3.8
						
Father's education						
Low	2155	13.9	1		1	
Middle	5393	13.5	1.1	0.9–1.2	1.1	0.9–1.4
High	2464	10.8	**0.8**	**0.7–0.9**	1.1	0.9–1.4
						
Father's occupation						
Upper white collar	3724	12.1	1		1	
Lower white collar	2399	13.6	**1.2**	**1.0–1.4**	1.0	0.8–1.2
Farmer or forestry	668	9.3	**0.7**	**0.5–0.9**	0.9	0.6–1.2
Blue collar	3685	14.0	**1.2**	**1.0–1.4**	0.9	0.8–1.1
						
Mother's education						
Low	1499	14.2	1		1	
Middle	5171	13.8	1.0	0.9–1.2	1.1	0.9–1.3
High	3528	11.3	0.8	0.7–1.0	1.1	0.9–1.5
						
Factors describing the place of residence						
Urbanization level						
Capital city area	1441	11.5	1		1	
Cities	5543	15.3	**1.3**	**1.1–1.4**	1.2	0.9–1.6
Rural villages	2317	11.3	1.0	0.8–1.2	1.0	0.7–1.3
Sparsely populated areas	1254	8.2	**0.7**	**0.6–0.9**	0.9	0.6–1.2
						
Region						
Southern	3948	12.1	1		1	
Northern	1664	13.7	1.1	0.9–1.4	1.2	0.9–1.5
Eastern	1336	12.5	1.1	0.9–1.3	1.1	0.8–1.4
Western	3801	13.7	**1.2**	**1.0–1.3**	**1.2**	**1.0–1.5**

Statistically significant ORs are bolded

*Adjustment for age, survey year, age at menarche and the significant variables of the previous model.

In addition among girls aged 16–18 years, EC use was lower in girls who were at high school compared with those were not at school (OR adjusted for age and year = 0.7, 95% CI 0.5–0.9). However, the difference in EC use was not statistically significant after adjustment for other covariates.

In multivariate analysis after adjustment for all covariates significant in previous model as well as age and survey year, EC use increased by the level of alcohol consumption, daily smoking and dating. The higher the use of alcohol, the more common EC use was. Girls lived with one parent with or without a stepparent used EC more frequently than those lived with both parents. It was higher in the western than in the southern of Finland. The use of EC was not associated with educational career, school achievement, father's education and occupation, mother's education and urbanization level.

**Figure 1:**
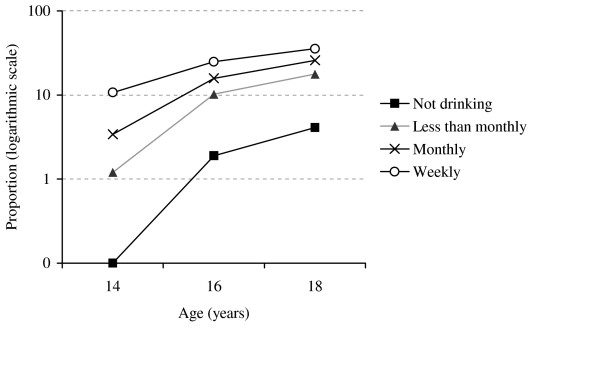
Proportion (%) of emergency contraceptive users according to alcohol consumption and age.

An interaction was found between age and alcohol consumption for the use of EC (p < 0.001) (Figure 1). The differences in the EC use between alcohol use groups were much higher at age 14 than at age 16 or 18. There was no interaction between age and survey year for EC unawareness or use.

When logistic regression models were run separately for 1999–2001 (prescription status) and 2003 (non-prescription status), the associations of individual, family and regional factors with EC use did not differ between these two periods.

## Discussion

Our findings demonstrate that the awareness of EC is high among Finnish adolescent girls. The use of EC did not increase after providing EC without prescription when compared to those years when prescription was needed. Alcohol consumption was strongly associated with higher EC awareness and use. The association of individual, family, and regional factors with EC awareness and use remained mostly stable during 1999–2003.

This study showed a high level of EC awareness. Public discussion in media in connection with the change to non-prescription status seems not to have increased it any more. The high level of EC awareness was shown already in 1996 in the Finnish School Health Promotion Study [9], which showed that more than 95% of 14–17 year-olds knew what emergency contraception is. In Finland, sex education has been an integral part of school curriculum, which probably explains partly the high level of awareness.

Teenagers should have knowledge of contraception, including EC, already before engaging in sexual relationships. EC is, first of all, a back-up method for those who use condoms as their contraception. Adolescents are an essential group of users, because condoms are a popular contraception at the early stages of sexual career. The present study showed that Finnish adolescents are highly aware of EC already many years before sexual relationships are topical; even two thirds of the youngest girls aged 12 knew about the method. However, only awareness of EC is not enough. There is a concern that although adolescents know about EC, they perhaps don't know how to obtain the pills and how to use them [18]. This kind of detailed knowledge could not be explored in our mailed survey, where the respondents could have checked the facts.

In our study, the variation of awareness by socio-economic characteristics of the family was not large while there were larger differences according to health behaviour. In line with other studies [6,19] we found a positive association between EC awareness and teenagers' school performance, which emphasizes the influence of educational, and sociodemographic factors. Unlike another study [6] we found no association between EC awareness and the level of father's education. A Swiss study showed a positive association with teens' scholastic curriculum so that girls with higher education are more aware of EC than those with mandatory school, and on girls whose fathers had higher education are more aware of EC than those whose fathers have low education [6].

We found no significant increase in EC use over time, while studies from other countries [7,20] have shown an increase in use over time. Indeed, there was no remarkable change in EC use after making EC available over the counter either. The results are consistent with the results of the Finnish School Health Promotion Study, which showed no change either among girls aged 14–18 years in 1996–2005 [21]. An explanation for the mild effect may be that access to emergency contraception was quite easy already before the non-prescription period. The Yuzpe method had been available since 1987, and it was reasonably easy to get a prescription from family planning clinics, school and student health services, and health centres [9]. Consistent with our study, making EC available over the counter did not lead to an increase in its use in Great-Britain [3]. On the other hand, a population-based study in Canada showed an increase of 55% in EC use in girls aged 15–19 years after allowing EC to be sold over the counter [4].

Concerning the minimal effect of use after the change to non-prescription status, some other explanations could be possible. First, the price of the product is relatively high, currently about 16–24 euros (20–30 US dollars) per one-time package. Perhaps teenagers most in need of this service cannot afford the product. If this is the case, the accessibility of EC has not actually improved despite the change to non-prescription status. One of the factors that have been described as limiting a more extensive use of emergency contraception is the fact that a large proportion of requests occur over the weekend, when the family planning clinics and most of the pharmacies are closed [19]. Furthermore, one year after making EC available over the counter is a short period to see the effect of non-prescription status. It may increase EC use in a longer period.

Some previous studies [4,6] have shown that teenagers in urban areas use EC more often than those in rural areas, which may reflect easier access to health services in urban areas. In the present study, the use of EC did not differ between urban and rural areas. A consistent result has been reported from Sweden [10], which like Finland is a large country with long distances to services in rural areas. However, equal access to services is an essential goal in health care, and arrangements in services have been carried out according to this principle.

An interesting finding was that the awareness of EC as well as its use was higher among those who used alcohol and smoked than among non-drinkers and non-smokers. This shows that those in need also know better and use them more often. Our finding is consistent with earlier studies [16,22,23] which have shown that smokers and alcohol consumers are at higher need of EC than non-smokers or non-drinkers. Smoking and alcohol drinking are associated with early sexual activity [24,25]. Moreover, teenage smokers or drinkers are even more likely to engage in risky sexual behaviour and have unprotected intercourse than non-smokers or non-drinkers [17,26]. A Swedish study [17] has reported that alcohol consumption is an important contributing factor for not using condom. Nonusers of condom therefore are more likely to take EC.

The current study was based on highly comparable national surveys, which have maintained similar data collection methods, samples and questions over the years. The repeatability of the questions on EC use and awareness was high. Unfortunately, questions on sexual behaviour could not be included in surveys posted to home, as this might have lowered the response rate. The analysis of the non-respondents indicated that the use of emergency contraception was higher among late responders aged 16 or 18 years. Therefore, non-respondents may have used more likely EC than responders, and we may have underestimated the proportion of EC use. We asked only about knowledge of the existence of the method and the results do not indicate that adolescents were well informed of details of its use.

In Finland, teenage sexual activity has increased between 1997 and 2001 [27] and abortion rate between 1994 and 2002 [28]. However, EC use did not increase and only a minimal increase was observed in the use of oral contraceptives during 1990–2003 [29]. It seems that despite the high level of awareness, EC is underused in Finland. This may have contributed to the rising trend in teenage abortions in Finland after the mid-1990s, together with insufficient use of regular contraception. Further research is needed to follow trends in the use of EC and examine the role that EC plays in the total contraceptive behavior of adolescents. Knowledge of EC among boys and their role in the use of the method are poorly understood and are considered important targets for further research.

## Conclusion

The awareness and use of EC did not increase after providing EC without prescription. Alcohol consumption is strongly associated with EC awareness and use. The characteristics of EC awareness and use remained stable over time.

## Abbreviations

EC emergency contraception

CI confidence interval

OR odds ratio

## Competing interests

The author(s) declare that they have no competing interests.

## Authors' contributions

AR designed and conducted the study. KFH and RS performed the statistical analyses. All authors interpreted the results. KFH drafted the manuscript. AR and EK critically revised the manuscript. All authors read and approved the final manuscript.

## Pre-publication history

The pre-publication history for this paper can be accessed here:


